# Correction: Utilization of Artificial Intelligence for the automated recognition of fine arts

**DOI:** 10.1371/journal.pone.0318114

**Published:** 2025-01-23

**Authors:** Ruhua Chen, Mohammad Reza Ghavidel Aghdam, Mohammad Khishe

The funding statement for this paper is incorrect. The correct funding statement is as follows: This research was supported by the Shanghai Culture and Education Integration Project (China) and the Ideological and Political Demonstration Course of the Ministry of Education (China).

The authors and PLOS One editors wish to inform readers that reference 6 of [1] was retracted on 10 August 2024 [2,3], while this article [1] was under review. The retracted article [2] was cited in the introduction, and it’s retraction does not appear to impact the reliability or validity of this article [1]. We reject that this issue was not identified prior to publication.
