# Supplementary material for: Utilization of Artificial Intelligence for the automated recognition of fine arts
Source: PLoS One. 2024 Nov 25;19(11):e0312739. doi: 10.1371/journal.pone.0312739 (PMC11588251; doi:10.1371/journal.pone.0312739)
Supplement: Attachment — Submitted filename: Response to the reviewers.pdf [file pone.0312739.s001.pdf]

Response to the reviewers

To: PLOS ONE
Original Manuscript ID: PONE-D-24-17832
Original Article Title: "Utilization of Artificial Intelligence for the Automated Recognition of Fine Arts"

Reviewer 1

Reviewer Point P 1.1 — Abstract: Should be restructured.

Author response:
Thank you for the reviewer's valuable feedback. We have restructured the abstract accordingly.

Author action: Here is the restructured abstract:
The recognition of fine art, traditionally dependent on human expertise, is undergoing a significant transformation with the integration of Artificial Intelligence (AI) and deep learning. This article introduces a novel AI-based approach for fine art recognition, utilizing Convolutional Neural Networks (CNNs) and advanced feature extraction techniques. Addressing the inherent challenges within this domain, we present a systematic methodology to enhance automated fine art recognition. By leveraging critical dataset characteristics such as objective type, genre, material, technique, and department, our method exhibits exceptional performance in classifying fine art pieces across diverse attributes. Our approach significantly improves accuracy and efficiency by integrating advanced feature extraction techniques with a customized CNN architecture. Experimental validation on a benchmark dataset highlights the efficacy of our method, indicating substantial contributions to the interdisciplinary field of fine art analysis.

Reviewer Point P 1.2 — Introduction: Latest literature should be cited.

Author response:
Thank you for your comment. I appreciate your feedback. I have now updated the introduction to include references to the latest literature:
In [16], the potential of established machine learning techniques to distinguish between authentic and imitation Pollocks is explored. A novel image ingestion method is developed, achieving 98.9% accuracy by decomposing images into multi-scaled tiles and utilizing transfer learning. Reference [17] explores fine-tuning CNNs for art-related image classification tasks, achieving state-of-the-art artist, genre, style, period, and national artistic context classification results. In [18], the focus was on advancing deep learning systems for automatic style classification by employing seven pre-trained EfficientNet models and custom architectures. The authors in [19] proposed a feature fusion method that combines various features used in painting captioning. The ablation study demonstrates that their feature fusion method enhances the model's performance by an average of 9.8%.
Please let me know if any further revisions are needed.
References:
16. Smith JH, Holt C, Smith NH, Taylor RP. Using machine learning to distinguish between authentic and imitation Jackson Pollock poured paintings: A tile-driven approach to computer vision. PloS One. 2024;19(6):e0302962.
17. HuW,LiX,LiC,LiR,JiangT,SunH,HuangX,GrzegorzekM,LiX. A state-of-the-art survey of artificial neural networks for whole-slide image analysis: from popular convolutional neural networks to potential visual transformers. Computers in Biology and Medicine. 2023;161:107034.
18. Menai, Baya Lina. Recognizing the artistic style of fine art paintings with deep learning for an augmented reality application. PhD thesis, Universite Mohamed Khider (Biskra-Algerie), 2023.
19. Yue Lu, Chao Guo, Xingyuan Dai, Fei-Yue Wang. Generating emotion descriptions for fine art paintings via multiple painting representations. IEEE Intelligent Systems. 2023;38(3):31–40.

Author action:

We updated the manuscript by adding the following:

1- In [16], the potential of established machine learning techniques to distinguish between authentic and imitation Pollocks is explored. A novel image ingestion method is developed, achieving 98.9% accuracy by decomposing images into multi-scaled tiles and utilizing transfer learning. Reference [17] explores fine-tuning CNNs for art-related image classification tasks, achieving state-of-the-art artist, genre, style, period, and national artistic context classification results. In [18], the focus was on advancing deep learning systems for automatic style classification by employing seven pre-trained EfficientNet models and custom architectures. The authors in [19] proposed a feature fusion method that combines various features used in painting captioning. The ablation study demonstrates that their feature fusion method enhances the model's performance by an average of 9.8%.

2- New References:

16. Smith JH, Holt C, Smith NH, Taylor RP. Using machine learning to distinguish between authentic and imitation Jackson Pollock poured paintings: A tile-driven approach to computer vision. PloS One. 2024;19(6):e0302962.

17. HuW,LiX,LiC,LiR,JiangT,SunH,HuangX,GrzegorzekM,LiX. A state-of-the-art survey of artificial neural networks for whole-slide image analysis: from popular convolutional neural networks to potential visual transformers. Computers in Biology and Medicine. 2023;161:107034.

18. Menai, Baya Lina. Recognizing the artistic style of fine art paintings with deep learning for an augmented reality application. PhD thesis, Universite Mohamed Khider (Biskra-Algerie), 2023.

19. Yue Lu, Chao Guo, Xingyuan Dai, Fei-Yue Wang. Generating emotion descriptions for fine art paintings via multiple painting representations. IEEE Intelligent Systems. 2023;38(3):31–40.

Reviewer Point P 1.3 — Results and discussion: Some grammatical mistakes are there which should be removed.

Author response:

Thank you for your comment. We have reviewed and corrected any mistakes to ensure clarity and precision in our presentation of the results.

Reviewer Point P 1.4 — Conclusion should be revised.

Author response:

Thank you for your comment. We have revised the Conclusion to improve its clarity.

Author action:

We updated the conclusion:

This paper rigorously explores automated fine art recognition methodologies, culminating in the introduction of an innovative approach designed to enhance accuracy and efficiency significantly. Our method harnesses critical dataset attributes such as objective type, genre, material, technique, and department, demonstrating exceptional performance in classifying fine art pieces across diverse attributes. Our approach showcases remarkable performance by integrating advanced feature extraction techniques and a tailored CNN architecture, substantially advancing automated fine art recognition. Furthermore, this technological leap holds promise for numerous applications, including art history research, cultural heritage preservation, and digital art curation. It highlights its profound impact on the intersection of technology and art appreciation. To comprehensively capture these advancements, future research could explore additional dimensions such as temporal analysis of artistic styles and further integration of interdisciplinary perspectives in art and technology.

Reviewer 2

Reviewer Point P 2.1 — The innovation of the article should be highlighted more. CNN networks are not a completely new type of network in the field of machine learning. The author mentioned improvements to CNN networks in the article. Therefore, the comparison between traditional CNN networks and improved CNN networks in art recognition accuracy should be increased, not just with algorithms such as BN, KNN, SVM, etc.

Author response:
Thank you for your valuable and constructive feedback. We appreciate your suggestion to highlight the innovation of our approach and provide a more comprehensive comparison with traditional CNN networks and improved versions in art recognition accuracy.

Author action:
We updated the manuscript by adding the following:
1- We have emphasized the innovation of our approach, particularly focusing on enhancements made to CNN architectures specific to fine art recognition.
2- We have conducted additional experiments to compare the performance of traditional CNN networks with our improved CNN networks in art recognition accuracy and specificity. These results are presented in Table 6 and Figure 5 for direct comparison and evaluation.
3- " For example, the accuracy results for our proposed method and the traditional algorithms—CNN, BN, KNN, SVM, and RF—are 93.24%, 93.34%, 90.58%, 78.37%, 75.41%, 83.27%, and 87.63%, respectively. Overall, our proposed CNN model outperforms all traditional classification models." is added.
This action plan addresses the reviewer's suggestion by highlighting the innovation of your approach and providing a comparative analysis between traditional and improved CNN networks in the context of art recognition accuracy.

Reviewer Point P 2.2 — There are some issues with inconsistent image clarity in the manuscript, such as Figures 3 and 5. The clarity of the coordinate axis urgent legend in Figure 3 is too low.

Author response:
We appreciate the reviewer's observation and feedback. Ensuring clear and understandable visual presentation is crucial to communicate our findings effectively. We have taken steps to improve the quality of these figures.

Author action:
We updated the manuscript by adding the following:
1- Enhanced the clarity of Figures 3 and 5 by improving the resolution of the coordinate axis and legends, ensuring better readability.
2- Revised the legends in Figure 3 to ensure they are clear and legible, addressing the specific concerns raised by the reviewer.

Reviewer Point P 2.3 — The conclusion of this manuscript is too simplistic and should be more comprehensive.

Author response:
We appreciate the reviewer's observation and feedback. We have revised the Conclusion to improve its clarity.

Author action:
We updated the conclusion:
This paper rigorously explores automated fine art recognition methodologies, culminating in the introduction of an innovative approach designed to enhance accuracy and efficiency significantly. Our method harnesses critical dataset attributes such as objective type, genre, material, technique, and department, demonstrating exceptional performance

in classifying fine art pieces across diverse attributes. Our approach showcases remarkable performance by integrating advanced feature extraction techniques and a tailored CNN architecture, substantially advancing automated fine art recognition. Furthermore, this technological leap holds promise for numerous applications, including art history research, cultural heritage preservation, and digital art curation. It highlights its profound impact on the intersection of technology and art appreciation. To comprehensively capture these advancements, future research could explore additional dimensions such as temporal analysis of artistic styles and further integration of interdisciplinary perspectives in art and technology.
